# Preparing Patients and Clinicians for Open Notes in Mental Health: Qualitative Inquiry of International Experts

**DOI:** 10.2196/27397

**Published:** 2021-04-16

**Authors:** Charlotte Blease, John Torous, Anna Kharko, Catherine M DesRoches, Kendall Harcourt, Stephen O'Neill, Liz Salmi, Deborah Wachenheim, Maria Hägglund

**Affiliations:** 1 Division of General Medicine Beth Israel Deaconess Medical Center Boston, MA United States; 2 Department of Psychiatry Beth Israel Deaconess Medical Center Boston, MA United States; 3 School of Psychology University of Plymouth Plymouth United Kingdom; 4 Department of Psychiatry Harvard Medical School Boston, MA United States; 5 Department of Women's and Children's Health University of Uppsala Uppsala Sweden

**Keywords:** open notes, electronic health records, attitudes, survey, mental health, psychiatry, psychotherapy, qualitative research, mobile phone

## Abstract

**Background:**

In a growing number of countries worldwide, clinicians are sharing mental health notes, including psychiatry and psychotherapy notes, with patients.

**Objective:**

The aim of this study is to solicit the views of experts on provider policies and patient and clinician training or guidance in relation to open notes in mental health care.

**Methods:**

In August 2020, we conducted a web-based survey of international experts on the practice of sharing mental health notes. Experts were identified as informaticians, clinicians, chief medical information officers, patients, and patient advocates who have extensive research knowledge about or experience of providing access to or having access to mental health notes. This study undertook a qualitative descriptive analysis of experts’ written responses and opinions (*comments*) to open-ended questions on training clinicians, patient guidance, and suggested policy regulations.

**Results:**

A total of 70 of 92 (76%) experts from 6 countries responded. We identified four major themes related to opening mental health notes to patients: the need for clarity about provider policies on exemptions, providing patients with basic information about open notes, clinician training in writing mental health notes, and managing patient-clinician disagreement about mental health notes.

**Conclusions:**

This study provides timely information on policy and training recommendations derived from a wide range of international experts on how to prepare clinicians and patients for open notes in mental health. The results of this study point to the need for further refinement of exemption policies in relation to sharing mental health notes, guidance for patients, and curricular changes for students and clinicians as well as improvements aimed at enhancing patient and clinician-friendly portal design.

## Introduction

### Background

A growing number of health organizations worldwide now offer patients web-based access to their clinical notes (*open notes*) [1]. Using secure internet portals, patients can log in and, at their own convenience, rapidly access and read their clinical documentation. Access is not just to lists of medications, laboratory results, referrals, or visit summaries but also to the very words written by clinicians. Emerging from the participatory design movement in Scandinavia [2]—often called *the Scandinavian Approach*—which strives for *democracy and democratization* in digital design [3], most patients in the Nordic countries are already offered open notes. Different cultural and policy considerations have advanced open notes in the United States; starting April 5, 2021 (postponed from November 2, 2020, owing to COVID-19), new federal rules mandate that, with few exceptions, all health providers must offer patients access to their web-based clinical notes [4,5]. The driving force behind these rules is the 21st-century Cures Act, which was enacted with the goal of accelerating medical product development and bringing innovations to patients quickly and efficiently [6].

However, sharing mental health notes, including psychiatry and psychotherapy notes, remains controversial, and some clinicians are uncertain about when it is appropriate to hide notes from patients. In Sweden, where the majority of patients have access to open notes (via *Journalen* the country’s eHealth portal), mental health notes are shared in psychiatric centers in 11 out of 21 regions of the country. In Norway, where all patients can access their clinical notes, a survey of psychiatry clinicians working in hospitals found that 8% kept a *shadow record* to prevent patients from reading all of their notes [7]. In the United States, psychotherapy notes are exempt from the new federal rules, and *information blocking* is permitted, if doing so “...will substantially reduce the risk of harm” to a patient or to another person [4]. Licensed health professionals can decide what constitutes a substantial risk “...in the context of a current or prior clinician-patient relationship” [4]. These rules leave considerable room for interpretation, and it is unclear how clinicians’ discretion will be monitored or evaluated.

Beyond policy and auditing considerations, surveys show that many mental health clinicians worry that patients will become anxious or confused after reading their notes [8,9]. In a study conducted at the US Veterans Health Administration, 63% (n=127) of clinician respondents described being less detailed in their documentation as a result of patient access and 49% (n=98) reported that they would be *pleased* if the practice was discontinued [9]. In Sweden, 62% (n=39) of clinical psychologists reported being less candid in their notes after the implementation of patient access to Journalen [8]. In lieu of adequate clinician training on writing mental health notes, ad hoc strategies aimed at minimizing patient harm, confusion, or disagreements with clinicians may undermine best practice.

Some surveys of patients’ experiences with reading their mental health notes are promising. For example, in a recent comparison of primary care patients with and without a mental health diagnosis, Klein et al [10] reported no differences in patient experiences with open notes: 92% (336) of patients with a mental health diagnosis compared with 91% (1789) of patients without a mental health diagnosis reported feeling more in control of their health care. A pilot study at an outpatient psychiatric clinic in Boston found that the majority of patients reported a better understanding of their mental health condition and better remembering their care plan [11]. However, not all patients report benefits from reading their mental health notes, and some studies suggest that patient trust may be enhanced or strained by access [12,13] Generally, research on opening notes with mental health patients is limited, and there is little discussion in the literature about how to provide patient guidance about the benefits and risks of accessing their clinical notes [14].

### Objectives

There is now extensive research on sharing outpatient visit notes with patients seeking medical care [15,16]. Although some surveys have examined mental health clinicians’ attitudes about open notes [9,11], only a few have been conducted among clinicians with experience of sharing their notes with patients [8,17]. Although in many countries, such as the United States, the majority of mental health care is provided in primary care, so far, few surveys have analyzed the experiences of open notes among mental health patients in that setting [10]. In addition, few surveys have solicited the experiences and opinions of patients who have read outpatient or inpatient psychiatry or psychotherapy notes [11,13,18,19]. Relatedly, we are not aware of any studies that have set out to explore the experiences with open notes of patients living with serious mental illnesses such as psychotic disorders, major depression, and bipolar disorders. Finally, only a limited number of investigations have examined how to prepare clinicians and patients for opening notes in the context of mental health care [14,20,21].

As previous publications have emphasized, open notes in mental health care do raise new practice dilemmas [18,22,23]. Clinicians must balance the duty to respect patient autonomy and transparent information disclosures while preventing the potential for patient harm from reading notes that may be upsetting or confusing [22,24]. Considering the pressing need for greater clarity about best practice in this domain, our goal was to initiate expert-led discussion on policy recommendations, including on how to better train clinicians and guide patients, for this practice innovation.

## Methods

### Background

We used a structured web-based survey to explore the consensus views of international experts. The qualitative web survey was embedded in a modified Delphi methodology structured around 3 rounds of surveys. The Delphi technique is an established methodology for exploring the consensus views of experts. It is especially well suited to forecasting in emergent areas of research and gauging opinions about new policies. This approach has also been applied extensively as a heuristic in health care management [25,26]. Experts are invited, in 3 rounds of polls, to give their anonymous opinions on a topic. Through an iterative process, the goal is to establish consensus opinions across the group.

Employing a purposive sampling methodology, the research team compiled a list of 92 participants with expertise in open notes in mental health. There is no universal agreement about the sample size for Delphi polls [27]; however, following previous surveys, our aim is to maximize the volume of responses balanced against maintaining high response rates between surveys [28,29]. As the survey was administered during the COVID-19 pandemic, it was uncertain how many responses we might obtain, and this factored into the decision to invite as many suitable experts as possible. The list was compiled after joint meetings in which the research team examined published research, gray literature, mass media articles, and personal connections to derive as inclusive a list as possible. Acknowledging the challenges associated with defining expertise in a given domain, we interpreted expertise as individuals who had experience, as clinicians, of sharing mental health notes with patients; patients with mental health diagnoses, including patient advocates and peers who had first-hand experience or knowledge of the practice; chief information officers, chief medical information officers, or directors of divisions of health organizations who had implemented sharing mental health notes; and informaticians and other health researchers, including patient researchers, who had published significant contributions within the field of open notes.

To ensure an international perspective, we specifically invited individuals from countries and health systems where clinical note sharing has been implemented. Measures were also taken to ensure gender, age, and demographic diversity. The study received ethical approval from the Beth Israel Deaconess Medical Center Institutional Review Board in April 2020 (reference number 2020P000218) and the University of Plymouth, United Kingdom. Invited participants were advised that the survey was confidential, and their identity would be restricted to a key member of the research team (AK). All the respondents provided informed consent before participating.

Prospective panelists were contacted via email in August 2020 with an invitation and internet link to the survey. Invitees were also informed that participation was voluntary, unpaid, and confidential to the survey team. Participants’ names were replaced with a study ID number by AK to preserve anonymity during data analysis.

### The Questionnaire

We created an electronic survey using JISC Online Surveys hosted by the University of Plymouth, United Kingdom [30]. The survey was conducted in English. Participants were sent 3 reminders, 1 week apart, and were given 4 weeks to respond to each round. The first round comprised questions about demographic information and the nature of participants’ expertise with open notes in mental health. This was followed by four sections with a total of 6 open-ended questions on sharing mental health notes and an additional open-ended question allowing participants to comment on the survey or submit additional responses (Textbox 1; Multimedia Appendix 1). The sections comprised (1) Effects on patients (2 open-ended questions), (2) Effects on clinicians (1 open-ended question), (3) Training and education (2 open-ended questions), and (4) Policy regulations (1 open-ended question). Responses to section 1 were used to form 2 additional rounds of the Delphi survey, and the results will be published elsewhere. In this study, we focused only on participants’ open-comment responses to sections 3 and 4, along with the response to the additional open-ended question.

**Round 1 open-ended questions.**

**Effects on patients**

- What, in your opinion, are the benefits, if any, of sharing mental health notes with patients?
- What, in your opinion, are the harms, if any, of sharing mental health notes with patients?

**Effects on clinicians**

- What, in your opinion, are the effects, if any, on clinicians of sharing mental health notes with their patients?

**Training and education**

- Should mental health clinicians be trained on how to write clinical notes for patients? If so, what should such training encompass?
- Should mental health patients receive guidance on how to read their mental health notes? If so, what should such guidance encompass?

**Policy regulations**

- What policy regulations, if any, should be in place for patient access to mental health notes?

**Comments**

- Do you have any other comments about sharing online mental health notes with patients?

### Qualitative Survey Component

Descriptive content analysis was used to investigate the responses using the following steps [31,32]. First, transcripts were read by CB, MH, and JT to familiarize themselves with responses. Second, a process was employed in which brief descriptive labels (*codes*) were applied to comments; for some comments, multiple codes were applied. This widely used method is considered an efficient methodology for qualitative data analysis [32]. Comments and codes were reviewed by CB, MH, and JT, with revisions leading to further refinement of the codes. Subsequently, first-order codes were grouped into second-order themes based on the commonality of meaning. CB, MH, and JT reviewed and refined the final themes.

## Results

### Overview

From a total of 92 experts from 6 different countries, 76% (70) of them responded. Among the 70 respondents, 50% (35) were female, and the mean age was 50 years (SD 11.52 years; Table 1). Of the 70 participants, 34% (24) had a PhD degree, and 64% (45) were currently working in clinical practice (Table 2). The mean number of years of experience working in health care, in open notes research, or as a patient advocate was 16 years. All respondents left comments (10,445 words), which were typically brief (1 phrase or 2 sentences).

**Figure 1:**
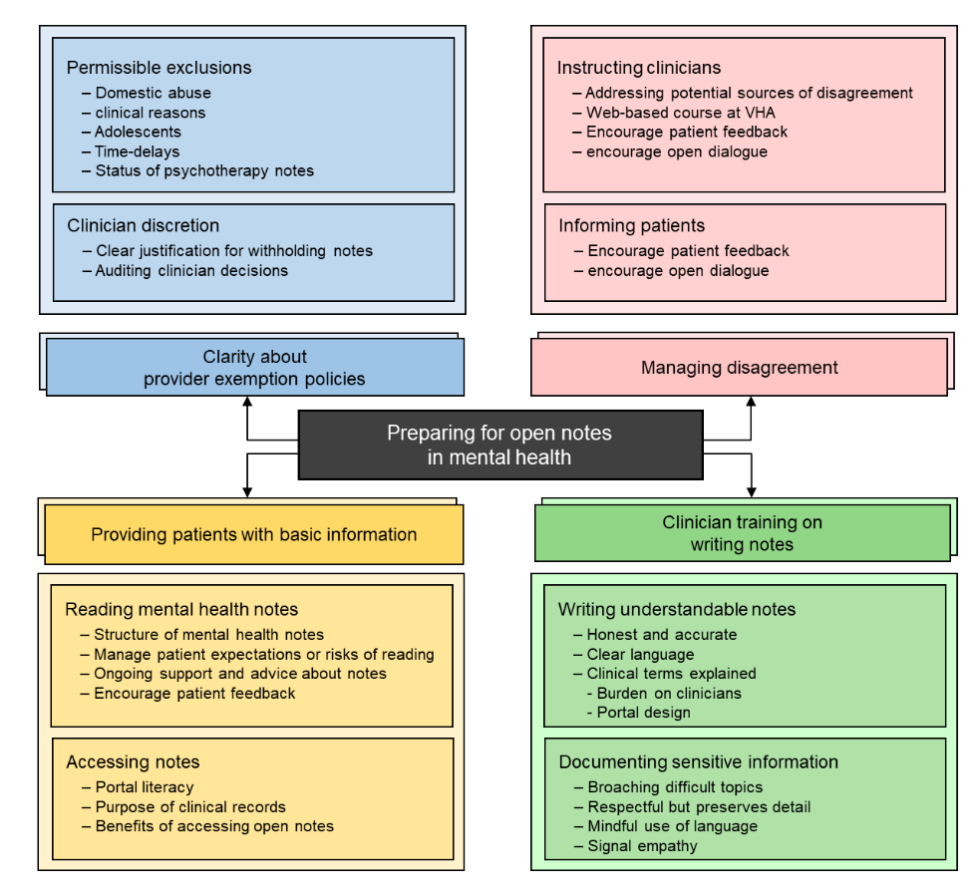
Themes and categories. VHA: Veterans Health Administration.

As a result of the iterative process of content analysis, participants’ comments on clinician training, patient guidelines, policy recommendations, and “any other comments” yielded distinctive themes. Owing to the limitations of the data set, these emergent themes reflected the topics of the questions and included (1) clarity about provider policies on exemptions, (2) providing patients with basic information about open notes, (3) clinician training in writing mental health notes, and (4) managing patient-clinician disagreements about mental health notes (Figure 1). These themes were further subdivided into categories, which are described below with illustrative comments.

**Table 1:** Demographic characteristics of respondents in round 1 (n=70).

Characteristic			Value^a^
**Gender, n (%)**			
	Female		35 (50)
	Male (including transgender male)		35 (50)
**Age (years)**			
	Mean (SD)		49.87 (11.52)
	**Age group (years), n (%)**		
		20-29	1 (1)
		30-39	19 (27)
		40-49	14 (20)
		50-59	19 (27)
		60+	17 (24)
**Ethnicity, n (%)**			
	Asian		6 (9)
	Black, African or Caribbean		1 (1)
	White		59 (84)
	Other		2 (3)
	Declined to answer		2 (3)
**Country of residence, n (%)**			
	Canada		3 (4)
	Estonia		1 (1)
	Norway		3 (4)
	Sweden		12 (17)
	United Kingdom		4 (6)
	United States		47 (67)

^a^Owing to rounding off, not all percentages may add to the total.

**Table 2:** Expertise of respondents in round 1 (n=70).

Characteristic			Value^a^
**PhD degree, n (%)**			24 (34)
	Biochemistry, cell biology or molecular pharmacology	3 (4)	
	Informatics (including eHealth, technology and health engineering)	9 (13)	
	Psychology (including biological and clinical)	8 (11)	
	Other (including economics, mathematics, medicine, and philosophy)	4 (6)	
**Works in a clinical practice, n (%)**			45 (64)
	Psychiatry (including adult, adolescent and child)	15 (21)	
	Primary care	10 (14)	
	Clinical psychology or psychotherapy	7 (10)	
	Psychiatric nursing	4 (6)	
	Pediatrics	3 (4)	
	Social work	3 (4)	
	Palliative care or home hospice	2 (3)	
	Peer support	2 (3)	
	Hospitalist	1 (1)	
	Radiology	1 (1)	
Experience of working in health care, open notes, or patient advocacy (years), mean (SD)			16.32 (12.23)
**Occupation or expertise related to health, open notes or patient advocacy, n (%)^b^**			
	Clinician	46 (66)	
	Researcher	25 (31)	
	Chief information officer or portal director or medical director	14 (20)	
	Patient advocate or person with lived experience	5 (7)	
	Social worker	1 (1)	

^a^Owing to rounding off, not all percentages may add to the total.

^b^Some participants indicated more than one area of expertise.

### Clarity About Provider Policies on Exemptions

#### Permitted Exclusions

Participants frequently expressed the view that exclusions to note sharing should be permissible in circumstances where access might lead to patient harm. Many comments referred to domestic abuse situations, for example, “notes that could endanger patients if read by eg, a relative/spouse.” As one participant noted:

If the relative forces the patient to log in to the portal, just the sight of mental health notes/visits might raise suspicion that the patient has been “gossiping.”

The view that exclusions should be permitted if there was “a specific clinical reason” was also commonly voiced, and participants offered suggestions for when hiding notes might be appropriate. One participant suggested that exemptions from sharing should be permitted among “patients with diagnoses or conditions that they believe will be destabilizing for the patient.” Other respondents offered more specific clinical scenarios or conditions, including “sexual trauma notes,” persons with “delusional” symptomatology, those with “multiple personality disorder, bipolar types I & II, schizophrenia, and active suicidal ideation,” and persons with “psychosis, personality disorders, and substance abuse disorders.”

In other cases, such as access to notes by adolescents, a few participants expressed reservations or uncertainties about suitable policies and restrictions. As one expert commented:

What should be done with adolescents and their parents’ access to these notes?

Offering a different perspective, a few participants proposed the broader view that there should be, “exclusion of all psychotherapy notes” from mandatory sharing. However, several respondents remained neutral about opening mental health notes, asserting, “we need to do some more research,” and “the data needs to be collected to document the reality of benefits and impacts.”

Expanding on the notion of patient exclusions, some participants proposed that time delays to sharing mental health notes should be “temporarily possible” or that “therapy notes may need to be held longer before sharing.” One participant suggested:

There should be a delay period for mental health notes of at least a week.

Drawing on his or her experience, another respondent noted:

We have 12 years of experience of sharing clinical notes with mental health patients in Estonia. We use [an] opt-out concept in [our] nation-wide health information system. The law gives the physician the right to close patient data from the patient for up to 6 months. After that the notes should be disclosed.

Notably, however, not all participants agreed that exemptions to sharing mental health notes should be permitted and that “open notes means open notes.” Some comments reflected the view that restricting access would be unethical, for example:

I do not feel that it is ethically correct to set restrictions based solely on diagnoses.

Unilateral paternalistic decisions about what should or should not be disclosed to patients is not acceptable.

Anything short of real-time patient access and patient control of how and when they wish to access their own record demonstrates an inequity in patient-provider partnerships.

#### Clinician Discretion

Addressing the issue about when patient exclusions to opening mental health notes might apply, many comments conveyed the view that “clinicians should be given discretionary power to withhold part or all of the notes.” As another respondent noted:

I would advocate for clinician judgment always having the final authority to revoke or block notes from patients in very specific instances.

One respondent dissented from this position, noting:

I don’t like the idea of limiting the notes to certain patients because we then get into tricky ethical areas and subjectivity on the part of the clinician.

Elaborating further on the idea of discretionary judgments, however, some participants emphasized that additional measures must be in place. These comments emphasized that, with the responsibility of making decisions about when to block notes, the burden of justification for doing so should be on clinicians. For example:

The onus should be on the clinician to record why access should be restricted, giving a review date for when this restricted access should be reconsidered.

Several participants suggested that formalized checks and balances should be in place to confirm clinicians’ decisions about when to deny access to notes. For example:

there need[s] to be at least two clinicians signing off on that exclusion before it can be invoked.

One participant proposed the need for “regular audits and monitoring...to increase transparency of which patient records are blocked and why.” Going further, another participant suggested:

Organizations should be required to perform audits, by a panel of clinicians and paid service users, at intervals on: (a) the proportion of patients that are accessing their mental health records and organizations with very low rates of access should have their processes for access assessed; (b) the proportion of records that have restricted access and how much of that restricted access is past its “sell by date.”

### Providing Patients With Basic Information About Open Notes

#### Accessing the Notes

Basic issues such as patients’ digital literacy and their knowledge of using health portals formed an emergent category. For example:

[P]atients should receive information about how to actually reach the notes (the technical part, how to access the information).

Respondents also emphasized the need to communicate basic information about medical records; as one participant wrote:

Explain the purpose of the health record and mental health note.

Several comments also suggested that patients should be informed about the potential value of open notes, including “how it can lead to better outcomes” and how reading their notes can “inform treatment engagement and decisions.”

#### Reading Mental Health Notes

Multiple comments emphasized the importance of informing patients about how the notes were structured. Some participants suggested that such guidance should include “being given basic information about the mental state examination.” Another participant noted:

The mental status exam has caused some consternation amongst patients until it is explained to them.

Another common subcategory was preparing patients to manage their expectations about the content of notes, including “potential areas of friction or frustration;” “why a diagnosis is needed, and that this diagnosis may be fluid;” and reassuring patients that, “the note/diagnoses is not judgment (especially for substance use and personality disorders).” Many participants also described the importance of informing patients about the risk of being upset by what they read, for example:

Providing education about the potential emotional response to notes is critical.

Several respondents suggested that patients should be advised that they will be supported if they choose to read their notes. As one patient advocate noted:

I was a patient in a psychiatric ward for 20 years. I recently obtained all my notes. It is fascinating and also disturbing to see what professionals thought and said about me. It is important for patients to be supported while reading their notes, to prevent relapse or trauma.

Participants frequently noted that patients would need advice on how to raise questions about what they read in their notes. Some comments emphasized that patients should be “encouraged to bring questions” about their notes to clinicians.

### Clinician Training on Writing Mental Health Notes

#### Writing Understandable Notes

Participants commented on the need to train clinicians on “how to write so that a layman may understand the notes.” Multiple comments suggested that clinicians should also be advised on the “degree of medical jargon” that might be documented. Some respondents suggested that there should be a “reduction in jargon” or “avoidance of confusing medical jargon and abbreviations.” One participant went further by suggesting that clinicians should also become knowledgeable about common patient terminology:

There is an ever-growing list of colloquial expressions and a lexicon for describing diagnoses and symptoms that exists outside of the medical community. And sometimes it’s the same word a clinician would use but it means something totally different on a Reddit mental health forum.

Exploring another aspect of this category, multiple comments expressed the importance of training to preserve details in clinical documentation. Some stated that clinicians should not “dumb down” their notes that needed to be “accurate, objective, truthful.” One participant highlighted the need to instruct clinicians on the “documentation of uncertainty.”

In general, most comments suggested that the onus should be on clinicians to modify documentation practices, for example:

Clinicians should always be writing on the assumption that their audience has zero training...Effective communication is necessary for the job.

However, one respondent presented a portal design solution:

To solve the conflict between professional language precision, and lay person comprehension, one practical solution may be to embed a dictionary in the journal [medical records]. I have seen a proof of concept of such system. The patient was presented a journal text, and by hovering the mouse over a medical record, a dictionary box was presented.

#### Documenting Sensitive Information

One frequently identified predicament was the need for guidance on “how to describe sensitive matters” and “difficult topics,” including “how to manage situations/write content that may potentially be perceived in a negative light in a way that is clinically appropriate and respectful.” Some respondents emphasized that clinicians would need to be instructed that “important information should not be left out due to fear about the patient’s reaction.” As one participant noted:

a patient may discuss a sensitive issue that they do not want in the record and the clinician needs to know how to document information in a way that the patient is comfortable having in writing.

More generally, participants recommended that clinicians should be trained to adopt a “mindful approach” in documenting mental health notes and to use “patient-friendly” language. Respondents frequently emphasized that clinicians would require training in writing notes that were “not perceived as demeaning,” in adopting “less inflammatory terminology,” and in choosing language “to avoid stigma or embarrassment.” As one respondent noted:

[M]any patients stumble over “patient complains of” or “affect” or other common psych mental health terms.

One respondent suggested that it would be helpful for clinicians to be provided with:

[a] list of words that appear to be judgmental or offensive that are frequently used (eg, patient lied, patient was aggressive, patient denied, patient is non-compliant, patient was upset).

Participants also emphasized the need for training in writing notes with *empathy* and *warmth*. Some comments highlighted an opportunity to use notes to provide greater patient engagement and motivation for treatment goals. As one participant noted, training should also encompass “a framework that acknowledges strengths and doesn’t just pathologize;” or as another commented, “education should include an examination of patient strengths as well as deficits; too often MH [mental health] notes can be of the deficit model.”

### Managing Patient-Clinician Disagreement About Mental Health Notes

#### Instructing Clinicians

Another category of comments related to clinician training addressed potential disagreements arising from patient access to their mental health notes. As one participant remarked:

Clinicians should receive training on the ethics of sharing notes with patients and how to address conflicts.

Other comments suggested that training should encompass advice about how to discuss disagreements *in vivo* during visits, for example:

Such training is not about writing notes, but rather about how to communicate with patients about their illnesses in a way that promotes shared understanding and points of open disagreement (Rather than clandestine documentation).

Offering ideas on how to prepare clinicians for practice dilemmas, participants suggested the need to “provide tips, templates and scripts on how to address certain situations so clinicians feel more confident.” Several respondents cited “the web-based course from VHA [Veteran’s Health Administration]” as “a useful starting point” (a webinar on how to write mental health notes [20]). One participant noted the importance of patient feedback to improve writing notes:

when mental health clinicians are first learning how to write notes they should have patient evaluators. For more seasoned clinicians, a modified version of this training could take place.

#### Soliciting Patient Collaboration

Multiple comments also described the importance of providing advice to patients about what to do if they detected inaccuracies or omissions in their notes, including “how to handle anything they feel is factually incorrect or was misunderstood in terms of content.” Recommendations also encompassed perceived discrepancies in psychotherapy notes, for example, explaining to the patient that, “possible mismatches can always be sorted out during the next session.” Several participants noted the positive dimension of soliciting patient feedback. Some comments suggested that patients should be counseled that any errors or feedback present opportunities for working “collaboratively” and increasing “open dialogue between patient and provider.”

## Discussion

### Principal Findings

This qualitative study provides foundational cross-cultural insight into the views of a range of experts on open notes in mental health care. We identified 4 major categories related to opening mental health notes: (1) clarity about provider policies on exemptions, (2) providing patients with basic information about open notes, (3) clinician training in writing notes, and (4) managing patient-clinician disagreements.

Clarity about provider policies included both views that exclusions to note sharing should be permissible under certain circumstances and views that clinicians should be given discretionary power to decide when notes should be shared or not. However, these views were not fully agreed upon, as some participants stressed that restricting access would compromise clinicians’ ethical duties. Many respondents discussed special handling of notes around certain mental health conditions such as “psychosis, personality disorders, and substance abuse disorders,” among others. Although there is research on open notes and mental health, these conditions are often excluded in studies, resulting in a lack of knowledge. Patients with these disorders may react differently to mental health notes, but separating stigma and assumptions from facts is critical. For example, patients with psychosis are active users of mental health apps and are not paranoid about telehealth, despite common assumptions about their use of technology [33-35]. Open notes could make patients with psychosis paranoid; however, the opposite effect may be achieved. Immediate research is necessary to guide impending implementation efforts. As participants suggested, formalized monitoring is needed to confirm clinicians’ decisions and ensure that patients are not wrongfully denied access to their notes.

Although recent research suggests that mental health patients seek the same features on health system portals as medical patients [36], it must be acknowledged that people with mental health conditions, such as schizophrenia, are likely to have less access to portals and, thus, less ability to partake in open notes. At the same time, internet access through smartphones is increasing globally, and a recent study from Sweden indicated that 77% (n=11,001,189) of the visits to the national patient portal were made from a smartphone [37]. Ensuring easy mobile access to patient portals is an important means of increasing accessibility.

Supporting patients on how to access and read their mental health notes was a topic of less controversy. Participants suggested that patients may need basic information on how to access patient portals and notes and on the purpose and content of a health record and mental health notes. The proposed guidance included ways of preparing patients for what to expect when reading their mental health notes to reduce the risk of misunderstandings or harmful emotional responses. Similarly, the need for clinician training on writing notes was also agreed upon by most participants, including concrete advice on how to write more understandable notes and how to address sensitive topics. Participants agreed that information should not be left out of the record for fear of causing patient distress; however, clinicians should be guided on how to address these topics both in the conversation with the patient and in the note.

Disagreements between patients and clinicians caused by the notes were also described as an issue requiring more than just training in how to write better notes. Rather, notes should be seen as one component in the overall communication with the patient, and clinicians should be supported in how to address, and hopefully avoid, disagreement and conflict with patients both in the visit and through written communication. Patient feedback on note writing was highlighted as an important tool. In addition, participants also suggested providing patients with instructions or guidance on what to do when in disagreement with a note to facilitate dialogue and collaboration rather than conflict.

Finally, a topic that was not raised by the respondents in this study was differential diagnoses. In a recent survey of US physicians with experience of open notes, approximately 23% (n=176) of physicians reported changing how they wrote differential diagnoses [38]. The omission of answers focusing on this topic by our respondents could indicate that this is less of a consideration in mental health care. However, in a Swedish survey study, 1 in 5 (22%, n=147) mental health clinicians [8] admitted writing less candid notes, and in a US Veteran’s Health Administration study, the majority (69%, n=108) of mental health clinicians reported writing fewer details [9]. That open notes may have an impact on how notes are written seems clear, but further research is needed to further understand the types of changes and their consequences [39].

### Strengths and Limitations

This study provides a foundational qualitative exploration of expert opinions on open notes in mental health. Importantly, this inquiry builds on previous survey research conducted in this area by focusing on expert opinions, for the first time, on pressing questions about policy, clinician training, and patient guidance. The survey benefits from a wide range of expertise drawn from countries and health organizations where patients have access to their mental health notes. The international diversity and breadth of expertise of the respondents from 6 countries are major strengths of the survey. For web-based surveys, a 50% response rate for is considered high [40]; our survey secured a response rate of 76%, which was another major strength of the study.

This study has several limitations. Comments were brief—only 1 or 2 sentences or written in bullet points—restricting a more in-depth understanding of participants’ views. In addition, owing to the limitations of web-based surveys, it was not possible to probe or explore respondents’ comments to obtain a more in-depth understanding of their views. Although 11% (8/70) of the respondents identified as patients or patient advocates, we suggest future research should directly solicit the views of mental health patients as experts, particularly those with serious mental illness, who work outside of professional health care and academia.

In addition, most of our respondents were White and well educated, which may have affected their responses. Perhaps reflecting on this, questions about access to patient portals received fewer comments. Although this may have reflected the focus of the survey on mental health, many disadvantaged social groups (older patients, those with fewer years of formal education, and vulnerable patient populations) risk losing out on the benefits of access to their clinical notes [41]. For example, in the United States, not everyone has internet access or experience in the use of digital devices to be able to log on to health system portals or read their notes [42].

To address these limitations, further focus groups or interviews would help to facilitate a richer and more nuanced exploration of patients’ and mental health clinicians’ perspectives on the themes raised in this survey, and further qualitative research is warranted. Future research could aim to better understand the root causes of clinician omission of information, less candid notes, and writing fewer details. For example, there may be personal apprehension and fear by clinicians of increased workload, appointment duration, and/or being questioned or confronted. Such surveys or interviews might address clinician rationales that may not be widely or openly disclosed.

### Conclusions

The results of this study indicate that there is a need for training and support for both patients and clinicians regarding the practice of sharing mental health notes and that clear policies are needed to guide clinicians in the process of sharing and to ensure that patients are given access to their information.

We observe a tension in the results between complete transparency and clinicians’ need to be able to exclude certain information to prevent patient harm. Although participants generally favored note sharing in mental health care, some stressed the clinicians’ autonomy to make fine-grained decisions regarding what information to share, whereas others stressed patients’ rights to access all information and the ethical risks of leaving the decision to share to the individual clinician. Evidently, there is a need for evidence-based policies in this area. Surprisingly, few participants raised issues regarding the digital divide and the risk that some patients may not be able to access their notes, possibly indicating that there is still more focus on challenges regarding those patients who *do* access their notes.

The results of this study highlight the need for further training and support for both clinicians and patients regarding note sharing and more thoughtful refinements to user-friendly portal design. A major part of such training and support must address the need for culture change and a shift in mindset toward a more collaborative approach to patient-provider relationships. We suggest that priority should be on training clinicians, including students, on how to write mental health notes. There is a pressing need for transparent systems that support flexible and safe sharing of notes in mental health care. Engaging both patient advocates and eHealth design teams in this study is essential to forge innovative strategies that enable patient understanding of medical terms and feedback on notes, as suggested by the respondents. We hope that this study will provide both direction and inspiration for further research and policy reflection on patient access to their mental health notes.

### Ethical Approval

This study was deemed exempt by the Beth Israel Deaconess Medical Center Institutional Review Board on April 10, 2020 (reference number 2020P000218), and the University of Plymouth, United Kingdom.

## Appendix

Multimedia Appendix 1 Round 1 survey.

## References

[1] Essén A, Scandurra I, Gerrits R, Humphrey G, Johansen MA, Kierkegaard P, Koskinen J, Liaw S, Odeh S, Ross P, Ancker JS (2018). Patient access to electronic health records: differences across ten countries. Health Policy and Technol.

[2] Björgvinsson E, Ehn P, Hillgren PA (2010). Participatory design and "democratizing innovation". Proceedings of the 11th Biennial Participatory Design Conference.

[3] Gregory J (2003). Scandinavian approaches to participatory design. Int J Eng Educ.

[4] Health and Human Services Department (2020). 21st Century Cures Act: interoperability, information blocking, and the ONC health IT certification program. Federal Register.

[5] Blease C, Walker J, DesRoches CM, Delbanco T (2021). New U.S. law mandates access to clinical notes: implications for patients and clinicians. Ann Intern Med.

[6] Rucker DW (2020). Implementing the Cures Act — bringing consumer computing to health care. N Engl J Med.

[7] Kristiansen E, Johansen M, Zanaboni P (2019). Healthcare personnels’ experience with patients’ online access to electronic health records: differences between professions, regions, and somatic and psychiatric healthcare. Proceedings of the 17th Scandinavian Conference on Health Informatics.

[8] Petersson L, Erlingsdóttir G (2018). Open notes in Swedish psychiatric care (Part 2): survey among psychiatric care professionals. JMIR Ment Health.

[9] Dobscha SK, Denneson LM, Jacobson LE, Williams HB, Cromer R, Woods S (2016). VA mental health clinician experiences and attitudes toward OpenNotes. Gen Hosp Psychiatry.

[10] Klein JW, Peacock S, Tsui JI, O'Neill SF, DesRoches CM, Elmore JG (2018). Perceptions of primary care notes by patients with mental health diagnoses. Ann Fam Med.

[11] Peck P, Torous J, Shanahan M, Fossa A, Greenberg W (2017). Patient access to electronic psychiatric records: a pilot study. Health Policy Technol.

[12] Denneson LM, Chen JI, Pisciotta M, Tuepker A, Dobscha SK (2018). Patients' positive and negative responses to reading mental health clinical notes online. Psychiatr Serv.

[13] Cromer R, Denneson LM, Pisciotta M, Williams H, Woods S, Dobscha SK (2017). Trust in mental health clinicians among patients who access clinical notes online. Psychiatr Serv.

[14] Denneson LM, Pisciotta M, Hooker ER, Trevino A, Dobscha SK (2019). Impacts of a web-based educational program for veterans who read their mental health notes online. J Am Med Inform Assoc.

[15] Moll J, Rexhepi H, Cajander Å, Grünloh C, Huvila I, Hägglund M, Myreteg G, Scandurra I, Åhlfeldt RM (2018). Patients' experiences of accessing their electronic health records: national patient survey in Sweden. J Med Internet Res.

[16] Walker J, Leveille S, Bell S, Chimowitz H, Dong Z, Elmore JG, Fernandez L, Fossa A, Gerard M, Fitzgerald P, Harcourt K, Jackson S, Payne TH, Perez J, Shucard H, Stametz R, DesRoches C, Delbanco T (2020). Correction: Opennotes after 7 years: patient experiences with ongoing access to their clinicians' outpatient visit notes. J Med Internet Res.

[17] Petersson L, Erlingsdóttir G (2018). Open notes in Swedish psychiatric care (Part 1): survey among psychiatric care professionals. JMIR Ment Health.

[18] Strudwick G, Yeung A, Gratzer D (2019). Easy access, difficult consequences? Providing psychiatric patients with access to their health records electronically. Front Psychiatry.

[19] O'Neill S, Chimowitz H, Leveille S, Walker J (2019). Embracing the new age of transparency: mental health patients reading their psychotherapy notes online. J Ment Health.

[20] Dobscha SK, Kenyon EA, Pisciotta MK, Niederhausen M, Woods S, Denneson LM (2019). Impacts of a web-based course on mental health clinicians' attitudes and communication behaviors related to use of OpenNotes. Psychiatr Serv.

[21] Blease C, Torous J (2020). Opening mental health notes: 7 tips to prepare clinicians. Psychol Today.

[22] Blease CR, O'Neill S, Walker J, Hägglund M, Torous J (2020). Sharing notes with mental health patients: balancing risks with respect. Lancet Psychiatry.

[23] Blease C (2020). Are mental health patients entitled to see their medical notes?. Psyche.

[24] Blease CR, Walker J, Torous J, O'Neill S (2020). Sharing clinical notes in psychotherapy: a new tool to strengthen patient autonomy. Front Psychiatry.

[25] Donohoe H, Stellefson M, Tennant B (2013). Advantages and limitations of the e-Delphi technique. Am J Health Educ.

[26] Custer RL, Scarcella JA, Stewart BR (1999). The modified Delphi technique - a rotational modification. J Voc Tech Educ.

[27] Rubinson L, Neutens JJ (1987). Research Techniques for the Health Sciences.

[28] Norcross JC, Pfund RA, Prochaska JO (2013). Psychotherapy in 2022: a Delphi poll on its future. Prof Psychol Res Pr.

[29] Blease C, Kharko A, Locher C, DesRoches CM, Mandl KD (2020). US primary care in 2029: a Delphi survey on the impact of machine learning. PLoS One.

[30] Jisc online surveys.

[31] Mayring P (2014). Qualitative content analysis: theoretical foundation, basic procedures and software solution. SSOAR Open Access Respository.

[32] Braun V, Clarke V (2006). Using thematic analysis in psychology. Qual Res Psychol.

[33] Firth J, Torous J (2015). Smartphone apps for schizophrenia: a systematic review. JMIR Mhealth Uhealth.

[34] Torous J, Wisniewski H, Liu G, Keshavan M (2018). Mental health mobile phone app usage, concerns, and benefits among psychiatric outpatients: comparative survey study. JMIR Ment Health.

[35] Firth J, Cotter J, Torous J, Bucci S, Firth JA, Yung AR (2016). Mobile phone ownership and endorsement of "mHealth" among people with psychosis: a meta-analysis of cross-sectional studies. Schizophr Bull.

[36] Strudwick G, Booth RG, McLean D, Leung K, Rossetti S, McCann M, Strauss J (2020). Identifying indicators of meaningful patient portal use by psychiatric populations. Inform Health Soc Care.

[37] Hägglund M, Blease C, Scandurra I (2020). Mobile access and adoption of the Swedish national patient portal. Stud Health Technol Inform.

[38] DesRoches CM, Leveille S, Bell SK, Dong ZJ, Elmore JG, Fernandez L, Harcourt K, Fitzgerald P, Payne TH, Stametz R, Delbanco T, Walker J (2020). The views and experiences of clinicians sharing medical record notes with patients. JAMA Netw Open.

[39] Blease C, Torous J, Hägglund M (2020). Does patient access to clinical notes change documentation?. Front Public Health.

[40] Baruch Y, Holtom BC (2008). Survey response rate levels and trends in organizational research. Hum Relat.

[41] Blease C, Fernandez L, Bell SK, Delbanco T, DesRoches C (2020). Empowering patients and reducing inequities: is there potential in sharing clinical notes?. BMJ Qual Saf.

[42] Rodriguez JA, Lipsitz SR, Lyles CR, Samal L (2020). Association between patient portal use and broadband access: a national evaluation. J Gen Intern Med.

