# Supplementary material for: Preparing Patients and Clinicians for Open Notes in Mental Health: Qualitative Inquiry of International Experts
Source: JMIR Ment Health. 2021 Apr 16;8(4):e27397. doi: 10.2196/27397 (PMC8087962; doi:10.2196/27397)
Supplement: Multimedia Appendix 1 [file mental_v8i4e27397_app1.pdf]

Beth Israel Deaconess Medical Center

HARVARD MEDICAL SCHOOL TEACHING HOSPITAL

WELCOME

DELPHI CONSENSUS POLL
SHARING MENTAL HEALTH NOTES

- ROUND 1 -

OpenNotes®

PURPOSE

As a respected clinician, researcher, patient advocate and/or health administrator, you are invited to participate in this Delphi Poll as one of a panel of selected international experts on sharing mental health notes with patients.

The aim of this Delphi research poll is to gather international expert opinions about the practice of sharing mental health clinical notes with patients. Specifically, we are requesting your expert opinions about the possible benefits and harms of sharing mental health notes; training or education requirements needed for this practice; and any health policy considerations you consider important.

Your interest and responses are especially valuable at a time when patients are navigating the transition to telemedicine, and when access to mental health notes may be especially important to support patient care. In addition, many health care systems around the globe are moving toward greater transparency with the USA, as an example, requiring as of November, 2020 that all medical clinician notes be made

available on-line to patients.

Your answers will help to inform debates about clinical practice curricula, and policy issues, pertaining to the practice of sharing mental health notes with patients. We especially appreciate your input at this challenging time, when there is increased interest in clinical note sharing.

We thank all the experts who donate their time by responding to this Delphi Poll. We aim to make all recommendations/research that comes out of this work open access to our participants.

WHAT WILL PARTICIPATION ENTAIL?

This Delphi Poll will involve **three rounds** of surveys, taking around **5-10 minutes each**. During the next 3-4 months, we expect that participation will involve no more than 25 minutes of your time in total. Unfortunately, we cannot offer financial compensation for your time. However, we do appreciate your expert input.

WHO ARE WE?

We appreciate your interest in participating in this online research survey. We are a team of researchers based at OpenNotes, Beth Israel Deaconess Medical Center, Harvard Medical School, Boston, USA; Digital Psychiatry, Department of Psychiatry, Deaconess Medical Center, Harvard Medical School, Boston, USA; the Department of and Children's Health, Uppsala University, Sweden; and the School of Psychology, University of Plymouth, UK.

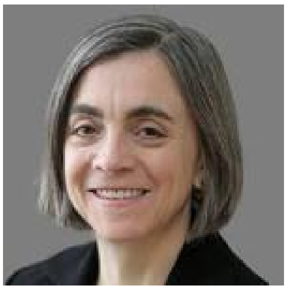

Dr. Catherine

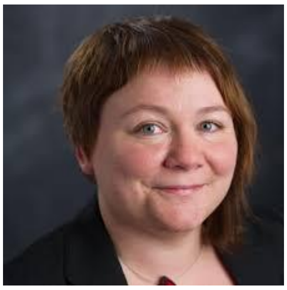

Dr. Maria Hägglund

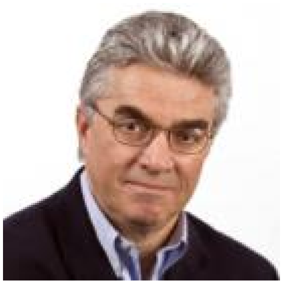

Dr. John Santa

DesRoches

OpenNotes

Harvard Medical School

OpenNotes

Uppsala University

Harvard

Medical School

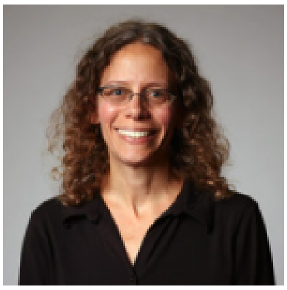

Deborah Wachenheim

OpenNotes

Harvard Medical School

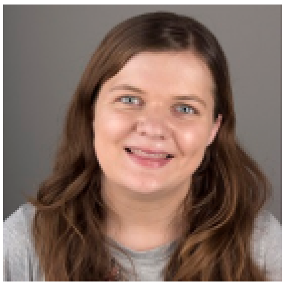

Kendall Harcourt

OpenNotes

Harvard Medical School

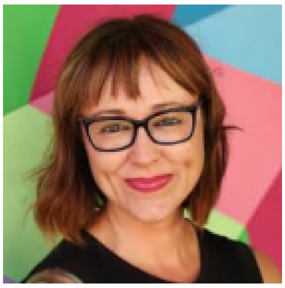

Liz Salmi

OpenNotes

Harvard Medical School

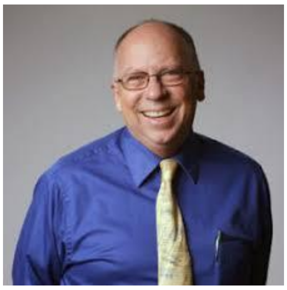

Dr. Stephen O'Neill

OpenNotes

Harvard Medical School

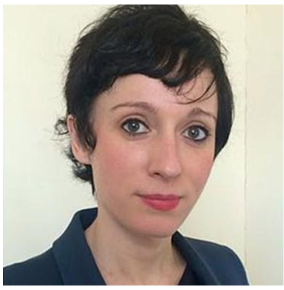

Dr. Charlotte Blease

OpenNotes

Harvard Medical School

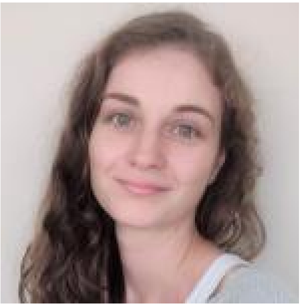

Anna Kharko

University of Plymouth

PREAMBLE

ABOUT THE STUDY

DESCRIPTION

This first round will consist of several open-ended questions. We are interested in your expert opinions about the practice of sharing online mental health notes with patients, including the benefits and harms, your opinions about education and training, and policies around this practice. In the first round we will also ask you some demographic questions, including about your professional background. **Round One should take around 10 minutes to complete.** Participants are requested to complete surveys in one sitting, and partial survey data will also be collected. **Round One will close at midnight EST on September 20th, 2020.**

The answers provided by Delphi panel members in the first round will be transformed into statements. These statements will be sent out anonymously in the second round, to assess consensus amongst expert opinion. In the third and final round, statements that did not reach consensus will again be sent out anonymously and you will be invited to amend your answer (should you wish to do so) against the average response of Delphi panelists.

Your answers will be confidential among selected members of the survey team, and anonymous to other participants.

TIME & COMMITMENT OF PARTICIPANTS

We will ensure that there is adequate time for panelists to provide their responses between rounds. Although this survey is not very demanding of time, the quality of Delphi Polls is dependent on a high level of participation between rounds. Therefore, should you be willing to participate, we request that you consider whether you can commit to responding to each of the three survey rounds.

All the data is completely anonymous. This study has been given ethical approval by Beth Israel Deaconess Medical Center, Boston, and the University of Plymouth, UK. The reference number for this study is Protocol #: 2020P000218.

DO I HAVE TO TAKE PART?

Participation is voluntary. You may withdraw at any point during the questionnaire for any reason, before submitting your answers, by closing the browser.

HOW WILL MY DATA BE USED?

The data we gather will be stored in a password-protected file and will be used to inform future academic publications. All questions are optional. The data will be stored for a minimum of ten years after publication or public release. Delphi panelists will also be asked whether they wish to remain anonymous or agree to their name being published in a journal article upon which the aggregate data will be based. It will not be possible to link data to individual participants.

WHO WILL HAVE ACCESS TO MY DATA?

Qualtrics (https://www.qualtrics.com/about/) is the data controller. You can read about their security policies here: https://www.qualtrics.com/security-statement/. The information will not be shared with anyone, and your name will be replaced by a numerical ID in all data analysis. Only selected members of the study team will be given access to the anonymous data for monitoring and/or audit of the study to ensure we are complying with guidelines, or as otherwise required by law.

WHAT IF THERE IS A PROBLEM?

If you have a concern about any aspect of this project, please speak to the PI Dr Cait DesRoches (cdesroch@bidmc.harvard.edu) telephone 001.617.975.7612, or Dr Charlotte Blease (cblease@bidmc.harvard.edu) who will do their best to answer your query. The researcher should acknowledge your concern within 10 working days and

give you an indication of how they intend to deal with it. If you have any complaints about this survey, please contact HSPO on 001.617.975.8500

By signing this form, you agree that you have read and understood your rights and provide consent to participate in this research.

- ◯ I have read and understood the study information and give my consent to continue.
- ◯ I do not wish to continue.

ABOUT YOU

ABOUT YOU

First Name & Last Name

[ ]

May we use your name in a potential academic publication?

- ◯ Yes
- ◯ No

Email

Gender

- Female
- Male
- Not listed (please specify)

Year of Birth

Country of Residence

Which of these best describes your ethnic group? (optional)

- Arab
- Asian

- Black / African / Caribbean
- Hispanic
- White
- Mixed / Multiple ethnic groups
- [ ] Other

MORE ABOUT YOU

MORE ABOUT YOU

Do you hold a PhD?

- Yes
- No

Please specify PhD subject area.

[ ]

Do you have a clinical specialty?

- Yes

- No

Please specify.

- Primary care
- Pediatrics
- Gerontology
- Clinical psychology
- Mental health nursing
- Psychotherapy
- Other

Do you currently work in clinical practice?

- Yes
- No

Please specify you clinical practice occupation
E.g. nursing, psychotherapy, clinical psychology, social work, psychiatry, primary care, etc.

What year did you begin working in health / OpenNotes / patient advocacy?

Current occupation or expertise in relation to sharing or accessing clinical notes

EFFECTS ON PATIENTS

EFFECTS ON PATIENTS

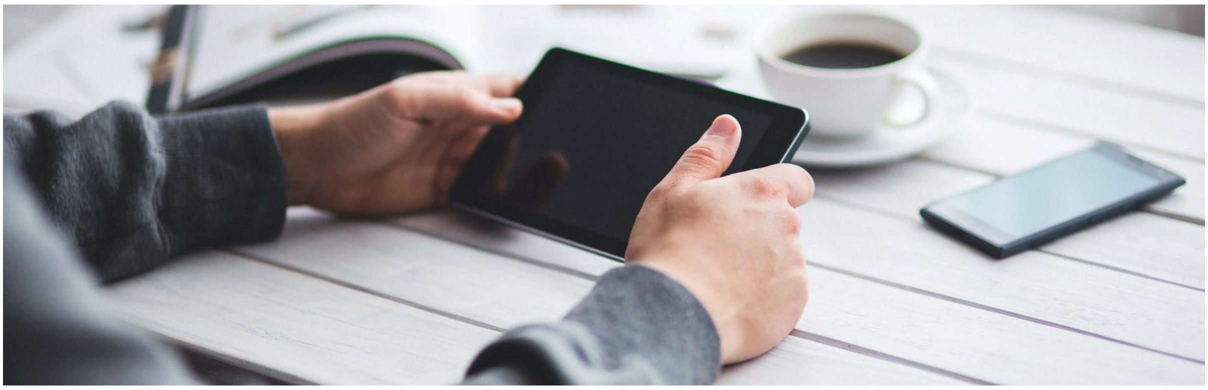

The following questions ask for **your expert opinions** about patients receiving online access to their mental health notes. We interpret 'mental health notes' to refer to any clinical notes written about a patient's mental health including notes written in psychiatry, clinical psychology, social work, psychotherapy contexts.

What, in your opinion, are the benefits - if any - of sharing mental health notes with patients?
Please briefly list at least 2 specific points.

What, in your opinion, are the harms - if any - of sharing mental health notes with patients?
Please briefly list at least 2 specific points.

EFFECTS ON CLINICIANS

EFFECTS ON CLINICIANS

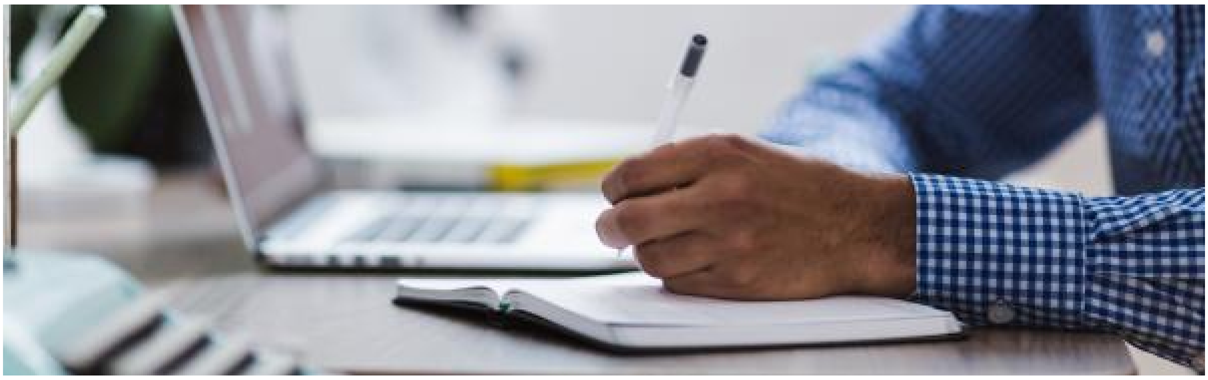

What, in your opinion, are the effects - if any - on clinicians of sharing mental health notes with their patients?
Please briefly list at least 2 specific points.

TRAINING & EDUCATION

TRAINING & EDUCATION

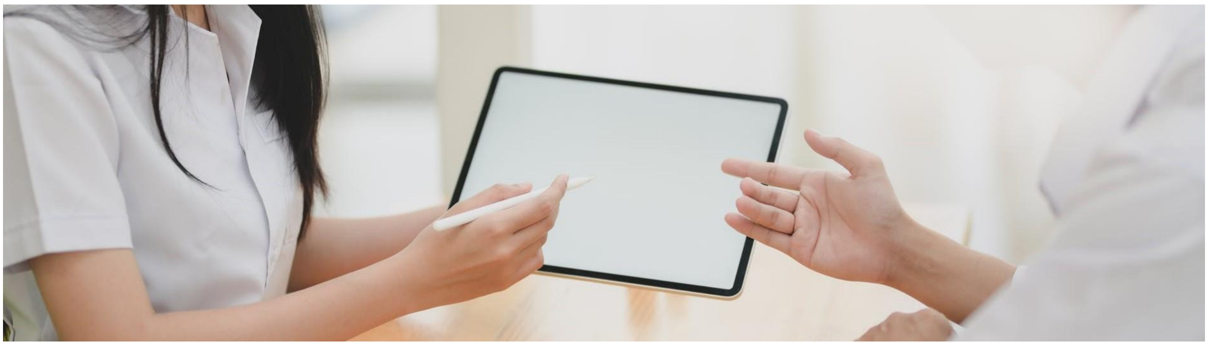

Should mental health clinicians be trained on how to write clinical notes for patients? If so, what should such training encompass? *Please briefly list at least 2 specific points.*

Should mental health patients receive guidance on how to read their mental health notes? If so, what should such guidance encompass? *Please briefly list at least 2 specific points.*

POLICIES FOR SHARING MENTAL HEALTH NOTES

POLICY REGULATIONS

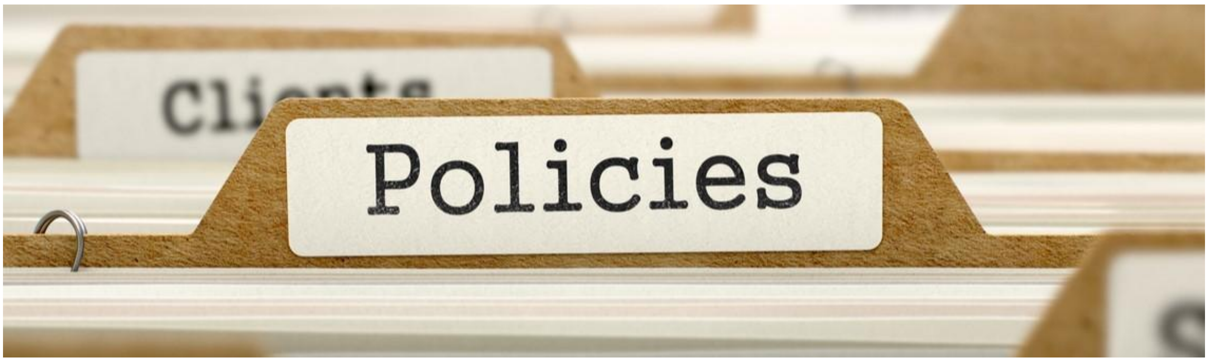

What policy regulations, if any, should be in place for patient access to mental health notes?

E.g. exclusion of some/all mental health patients? Exclusion of psychotherapy notes? Hiding the notes for a longer period of time? Mental health clinicians should be given discretionary powers about when to share notes?
Please briefly list at least 2 specific policy regulations.

COMMENTS

COMMENTS

Do you have any other comments about sharing online mental health notes with patients?

Powered by Qualtrics
